# Supplementary material for: Incorporating Cascade Effects of Genetic Testing in Economic Evaluation: A Scoping Review of Methodological Challenges
Source: Children (Basel). 2021 Apr 27;8(5):346. doi: 10.3390/children8050346 (PMC8145875; doi:10.3390/children8050346)
Supplement: Supplementary file 1 [file children-08-00346-s001.zip › children-1177038-supplementary.pdf]

SUPPLEMENTARY FILE A

How can methods for economic evaluation be modified to include cascade effects from genetic testing to improve the comprehensiveness and quality of evidence?

Search executed on Jan. 5, 2021.

Medline – 182 hits

exp cost-benefit analysis/ or exp health care costs/ or exp technology assessment, biomedical/ or exp models, economic/ or exp quality-adjusted life years/ or (cost-benefit analysis or health care cost* or biomedical technology assessment or economic model* or quality-adjusted life year* or cost analysis or cost-utility analysis or cost-consequence analysis or economic evaluation or health technology assessment or decision analytic model* or decision tree* or markov model* or microsimulation* or monte carlo simulation* or qaly or health outcome* or health effect* or resource utili* or resource use*).ab,ti

AND

(cascade or spillover* or spill over* or famil* or child* or parent* or mother* or maternal or father* or paternal or sibling or brother* or sister* or grandparent* or grandmother* or grandfather* or cousin* or first-degree relative or second-degree relative).ab,ti adj3 (effect* or cost* or consequence* or burden* or impact* or resource utili*).ab,ti

AND

exp genetic testing/ or exp sequence analysis, DNA/ or exp genetic predisposition to disease/ or exp genetic carrier screening/ or ((gen* or cascade or carrier* or famil* or prevent* or predict* or variant or mutation).ab,ti adj2 (test* or screen* or diagnos* or analys*).ab,ti)

Embase – 303 hits

exp cost benefit analysis/ or exp health care cost/ or exp biomedical technology assessment/ or exp economic model/ or exp quality adjusted life year/ or (cost-benefit analysis or health care cost* or biomedical technology assessment or economic model* or quality-adjusted life year* or cost analysis or cost-utility analysis or cost-consequence analysis or economic evaluation or health technology assessment or decision analytic model* or decision tree* or markov model* or microsimulation* or monte carlo simulation* or qaly or health outcome* or health effect* or resource utili* or resource use*).ab,ti

AND

(cascade or spillover* or spill over* or famil* or child* or parent* or mother* or maternal or father* or paternal or sibling or brother* or sister* or grandparent* or grandmother* or grandfather* or cousin* or first-degree relative or second-degree relative).ab,ti adj3 (effect* or cost* or consequence* or burden* or impact* or resource utili*).ab,ti

AND

exp genetic screening/ or exp sequence analysis/ or exp genetic predisposition or ((gen* or cascade or carrier* or famil* or prevent* or predict* or variant or mutation).ab,ti adj2 (test* or screen* or diagnos* or analys*).ab,ti)
